# Automating Rule-Compliant and Equitable Call Schedules for Orthopedic Surgery Residents With Artificial Intelligence and Large Language Models: A Simulation-Based Validation Study

**DOI:** 10.1177/23821205261441470

**Published:** 2026-04-12

**Authors:** Prushoth Vivekanantha, Marc Daniel Bouchard, Jeffrey Kay, Darren de SA, Olufemi R. Ayeni

**Affiliations:** 1 Division of Orthopaedic Surgery, 574408 Department of Surgery, 3710 McMaster University, Hamilton, ON, Canada

**Keywords:** automation, administration, scheduling, artificial intelligence, large language model, education, medical, graduate, health personnel

## Abstract

**Background:**

Call schedule generation is a time-intensive administrative task for residency programs. Traditional manual approaches often require hours of computation and can be inflexible. Large language models (LLMs) offer an efficient and adaptable alternative, therefore the purpose of this study was to assess if generative pretrained Transformer 5.2 (GPT-5.2, OpenAI) combined with a deterministic Python rule-checker, can automate complex, rule-compliant, and equitable call schedules for orthopedic surgery residents.

**Methods:**

Ten month-long residency blocks from a single institution were modeled, including both nonbackup and backup months in which junior residents required senior resident coverage. GPT-5.2 was accessed via the OpenAI application programming interface and prompted to follow 14 scheduling rules reflecting local institutional policy. For each block, 3 consecutive schedules were attempted, yielding 30 total runs. Performance was assessed by the proportion of successful, rule-compliant schedules generated and fairness metrics (Jain and Gini indices). Efficiency metrics included total wall-clock time, attempt duration, and estimated cost.

**Results:**

For nonbackup blocks, all 15 runs (100%) produced rule-compliant schedules with no terminations. Mean (SD) Jain and Gini indices were 0.948 (0.024) and 0.119 (0.031), respectively. Mean (SD) wall-clock time was 236 (148) s, with a mean (SD) cost per run of $0.15 ($0.03) United States Dollars (USD). For backup blocks, 13 of 15 (86.7%) runs produced successful, rule-compliant schedules, however, all blocks produced at least 2 valid schedules. Mean (SD) Jain and Gini indices were 0.936 (0.025) and 0.132 (0.031). Mean (SD) wall-clock time was 448 (400) s and the mean (SD) cost per run was $0.24 ($0.08) USD.

**Conclusion:**

GPT-5.2 can automate the generation of complex, rule-compliant and equitable call schedules for orthopedic surgery residents within minutes at a low computational cost of less than $1.00 USD.

## Introduction

Residency scheduling is a time-intensive administrative task that requires balancing multiple objectives: ensuring adequate coverage clinically, maintaining fairness and satisfaction among trainees, and adhering to institutional policies and duty restrictions.^1,2^ Often this task is performed by chief residents or program coordinators and can take many hours to complete.^2^ Furthermore, repeated revisions are often required to make adjustments in a “trial and error” fashion to accommodate vacation requests, modified service needs, and trainee preferences.^3^ Previous studies have suggested that administrative burden and scheduling contribute meaningfully to the stress and workload experienced by residents, highlighting the need for tools to automate this process.^4,5^

Classic operations-research methods have been used to automate schedule generation within healthcare, including for scheduling physicians.^3^ While these approaches can achieve solutions that follow all rules, they require highly complex mathematical formulation of each institutional rule and constraint,^6^ thus necessitating extensive reprogramming for every new context. Additionally, while these solutions do reduce the amount of manual burden, they can still result in computer run-times along the scale of hours. Generative artificial intelligence (AI), particularly large language models (LLMs) are increasingly being used for the automation of routine administrative tasks, and may serve as an even stronger option.^7-10^ LLMs are driven by advancements in deep learning and natural language processing (NLP) and specialize in general text summarization, interpretation, and generation.^7^

Despite the surge of research involving LLMs, there have been a limited number of studies in the literature that validated the use of this technology in scheduling residents for call ensuring that generated outputs follow a set of highly complex rules mimicking real life. LLMs allow for easier encoding of institutional rules directly within the text provided in a prompt, as opposed to modifying hard code, offering transformational potential in the way scheduling is performed in residencies. Therefore, the present study aimed to assess if generative pretrained Transformer 5.2 (gpt-5.2) combined with a deterministic Python rule-checker, can automate complex, rule-compliant, and equitable call schedules for orthopedic surgery residents. Specifically, the efficacy (success rate and fairness metrics) and efficiency (total time and cost) were assessed across multiple simulated blocks of differing complexity. It is hypothesized that a hybrid AI framework combining gpt-5.2 with a deterministic rule-checker could generate rule-compliant and equitable orthopedic resident call schedules with high success rates, minimal retries, and low computational cost.

## Methods

### Context, Sites, and Block Selection

Ten 28 day-long blocks were modeled after typical scheduling requirements, resident numbers, and vacation or accommodation requests from 3 hospital sites within a single Canadian orthopedic surgery residency program (academic years 2024-2025 and 2025-2026). No real resident information was used. Blocks were sampled into 2 categories: 5 “nonbackup” months in which no junior residents required additional coverage by a senior resident, and 5 “backup” in which junior residents required designated senior backup. For this analysis, juniors were defined as either postgraduate year 1 (PGY1) or PGY2, and seniors as PGY3 to PGY5. To reflect real-life practice from the institution, some blocks included a resident on a “research block” with a predefined maximum on total call assignments (*n* = 3), and some blocks included “fly-in” coverage (coverage by residents from another hospital site) on specific dates that did not require local scheduling. Ethics approval was not required for this study as it was simulation-based and did not involve human participants, identifiable data, or biological materials.

### Scheduling Rules

Schedules were considered viable only if they satisfied prespecified rules, which reflect the program's standard call policies a priori following a meeting with chief residents and after review of standard Professional Association of Residents of Ontario guidelines. These rules were as follows:

1. Avoid scheduling residents for calls during vacation/away dates and avoid local scheduling during “fly-in” coverage dates.
2. Residents should not be assigned on consecutive calendar days for either primary or backup call.
3. Each resident should only have a maximum of 2 weekend units. One weekend unit was considered as either a Saturday or Friday/Sunday pair. All residents (primary or backup) scheduled on a Friday must also be scheduled on the corresponding Sunday. Each resident should only have a maximum of 1 Saturday and 1 Friday/Sunday pair.
4. Each resident must have a total call count ranging from 2 to 7 (either primary or backup).
5. Research residents should only have a maximum of 3 calls per month.
6. All dates not having “fly-in” coverage must have a primary resident scheduled and a backup when a backup is needed.
7. Between all PGY cohorts, junior residents should either have more or the same amount of total calls as more senior residents.
8. Between all PGY cohorts, junior residents should have either more or the same amount of weekend units as more senior residents.
9. Within a specific PGY cohort, the difference in number of weekend units should be less than or equal to 1.
10. Within a specific PGY cohort, the difference in number of total calls should be less than or equal to 1.
11. Within a specific PGY cohort, the difference in total number of backup calls should be less than or equal to 1.
12. Between consecutive PGY cohorts, the maximum difference in total calls should be less than or equal to 2. For example, if a PGY2 has 5 total calls, the minimum number of calls that a PGY3 may have is 3. If no PGY3s are at the site, then this difference applies to PGY2s and PGY4s.
13. Research residents should not be included in rules 7 to 12 but they do apply for rules 1 to 6.
14. If a date's primary requires backup, a backup resident must be assigned from the eligible pool.

### Development of AI Model

A Python-based NLP workflow was built by the primary author to generate resident schedules from structured inputs and the above prespecified rules. The script ingested a json file input containing a roster (resident, PGY level, vacation or away dates, research status, if the resident needs backup, and if they are eligible for backup), and block details (start/end dates and fly-in dates). A rule-embedded prompt encoded all hard constraints (rule 1-14), which was then sent to the gpt-5.2 model (set on medium reasoning mode) (December 2025) via the OpenAI application programming interface (API). Assignment of calls then proceeded by following the multipass algorithm

Step 1: Assign Friday and Sunday primary calls as a unit, followed by Saturdays, filling from junior to senior (PGY1→ PGY5)

Step 2: Assign backups if needed for the weekends, starting from PGY3s to PGY5s

Step 3: Assign primary calls for weekdays starting from PGY1s to PGY5s

Step 4: Assign backup calls if needed for weekdays starting from PGY3s to PGY5s

Within the Python code, a deterministic script manually checked the schedule generated by gpt-5.2 ensuring that it followed all 14 rules. If the script detected a violation, the model was forced to restart and generate a new schedule. The model would be given 3 tries to generate a rule-compliant schedule before terminating. Furthermore, the model was given 500 s to generate a schedule. If it failed to generate a schedule within 500 s, the script timed out and was forced to restart. At 1500 s of wall-clock time, the script would retry 2 more times for a total of 4500 s before the script terminated.

For each of the 10 example blocks, the model was asked to generate 3 different schedules in succession. Therefore, there were a total of 30 separate runs (eg, 5 nonbackup blocks × 3 runs, 5 back-up blocks × 3 runs). The prompt utilized can be found in Table 1.

**Table 1.** Prompt Utilized for Schedule Generation.

"""You are an intelligent scheduling assistant. You schedule Orthopedic Surgery call. I/O – TZ: America/Toronto. Calendar dates are ISO (YYYY-MM-DD). – Input JSON keys (authoritative):
site: string block: string or int year: string or int start_date: YYYY-MM-DD (inclusive) end_date: YYYY-MM-DD (inclusive) fly_in_dates: [YYYY-MM-DD] residents: [ { name: str, pgy: 1–5, pgy_str: “PGY#”, backup_needed: bool, eligible_backup: bool, away_dates: [YYYY-MM-DD], research: bool },… ] Definitions – Weekend unit = Saturday OR (Fri/Sun pair). A Fri/Sun pair must be covered by the SAME Primary and SAME Backup. – No back-to-backs: no assignment on consecutive calendar days. – Away/Fly-in: cannot be assigned (any role). Fly-in date → Primary="Fly-in”. No Backup. – Backup: a second resident assigned to a date (if needed). – If a Primary has backup_needed = true, you must assign a Backup from residents where eligible_backup = true. – caps: { max_calls_per_resident:7, min_calls_per_resident:2, max_research_calls_per_resident:3}
HARD RULES 1) Respect away and fly-in dates. 2) No back-to-back (any role) on consecutive calendar days. 3) Weekend per-resident caps: ≤1 Saturday, ≤1 Fri/Sun pair, ≤2 total weekend units. 4) Each resident must have between caps.min_calls_per_resident and caps.max_calls_per_resident (primary + backup). 5) If research = true, total assignments ≤ caps.max_research_calls_per_resident. 6) Every non–Fly-in date needs a Primary; assign Backup when required and eligible exist (else leave blank and annotate Note). 7) Monotone totals by seniority (exclude research): for ANY junior cohort PGYj and ANY more senior cohort PGYk > j:
min_total(PGYj) ≥ max_total(PGYk). Ties allowed. 8) Monotone weekend units by seniority (exclude research residents from this rule): same pattern as #7 using weekend units. 9) Within-PGY WEEKEND balance (exclude research residents from this rule): range ≤ 1 if cohort has ≥2 non-research members. 10) Within-PGY TOTAL balance (exclude research residents from this rule): range ≤ 1 if cohort has ≥2 non-research members. 11) Within-PGY BACKUP balance (exclude research residents from this rule; only among eligible_backup = true): range ≤ 1 if ≥2 such residents. 12) Cross-cohort adjacency (exclude research residents from this rule): for adjacent present cohorts A (junior) and B (senior), for all a∈A, b∈B:
| total_calls(a) − total_calls(b) | ≤ 2. 13) Research exemptions for 7–12 (but still obey away/fly-in, no back-to-back, weekend caps; and max calls 3 by default). 14) If Primary.backup_needed = true, assign a Backup from eligible pool. If any eligible exist, leaving blank is a violation.
ALGORITHM (deterministic; no global backtracking) A) WEEKENDS-1: assign weekend primaries (Fri/Sun pairs as a unit, then Saturdays) from junior→senior (PGY1→PGY5). B) WEEKENDS-2: assign weekend backups where needed from PGY3→PGY5 (eligible only). C) WEEKDAYS-1: assign weekday primaries from PGY1→PGY5. D) WEEKDAYS-2: assign weekday backups where needed from PGY3→PGY5 (eligible only).
Output columns exactly: Date,Day of Week,Weekend Type (Sat/Fri-Sun/None),Primary Resident,Primary PGY,Backup Resident,Backup PGY,Notes If something is infeasible, leave the cell blank (don’t violate hard rules) and note why in Notes. “**"”**

### Outcomes

Both efficacy and efficiency metrics were assessed for each 30 runs within the 10 blocks. Efficacy metrics included the number of successful runs (success defined as a run that did not terminate due to 3 repeat rule-violation or timeouts and was able to generate a rule-compliant schedule) and overall fairness which was calculated using the Jain and Gini indices. The schedule for each resident was used to generate burden points based on the days that they were scheduled to be on call (weekday points = 1.0, Saturday = 1.5, Friday/Sunday = 1.5 points, with total points being the sum). Jain was defined as: (total summed burden)^2^/[*n*(sum of individual squared burdens)], where *n* = total number of residents. Values ranged from 0 to 1, with higher values indicating greater equity in shared burden among residents. Gini was defined as: (sum of absolute difference in burden between all pairs of residents within a block)/[2(*n*)(sum of total burden)] where *n* = total number of residents.^11,12^ Values ranged from 0 to 1, with lower values indicating more even sharing of burden among residents. Rule compliance was manually verified by the primary author.

Efficiency metrics included total wall-clock time, attempt time, and API cost. Wall-clock time was the full elapsed time from the start of the first attempt until a valid schedule was produced. Attempt time was the duration of the individual model call that successfully generated the final valid schedule. API cost was the estimated monetary cost based on the total based on the total number input and output tokens. This formula was estimated from the OpenAI website (# input tokens/1,000,000 × $1.75 United States Dollars (USD) + # output tokens/1,000,000 × $14 USD). Pricing can be found at https://openai.com/api/pricing/?utm_source = chatgpt.com. A token is the smallest unit of text that the model is able to process or generate.

### Statistical Analysis

Descriptive statistics were used to summarize efficacy and efficiency outcomes across all model runs. Continuous variables including Jain index, Gini index, wall-clock time, attempt time, and cost per run were reported as means with standard deviations and medians with ranges where appropriate. Categorical variables, including successful runs and retry events, were reported as counts and percentages. No other statistical testing was performed as the objective of the study was to evaluate feasibility across simulated scheduling runs than to compare groups.

## Results

### Nonbackup Blocks

Among the 5 nonbackup blocks, all 15 runs were successful in generating call schedules that adhered to all 12 rules (rules 11 and 14 do not apply to nonbackup scenarios). There were no retries due to noncompliant schedules, however there was 1 run that required a retry due to timing out at the 500 s mark. The median (range) Jain was 0.941 (0.088) with a mean (SD) of 0.948 (0.024). Furthermore, the median (range) Gini was 0.125 (0.117) with a mean of 0.119 (0.031).

The median (range) total wall-clock time was 195.03 (598.95) s while the mean (SD) was 236.19 (147.69) s. The median (range) time for individual successful attempts was 195.03 (185.21) s while the mean (SD) was 202.85 (52.93) s. The median (range) cost per run was $0.15 ($0.10) USD while the mean (SD) was $0.15 ($0.03) USD (Tables 2 and 3). All outputs can be found in the Online Supplemental Material.

**Table 2.** Summary of Efficacy Metrics Across Model Runs.

Category	Total Runs	Successful Runs (*n*, %)	Rules Checked	Jain Index (Mean ± SD)	Gini Index (Mean ± SD)	Retries From Noncompliance	Timeouts (*n*)	Notes
Nonbackup blocks	15	15 (100%)	12 (rules #12 and #14 only applicable to backup scenarios)	0.948 ± 0.024	0.119 ± 0.031	0	1	No rule violations
Backup blocks	15	13 (86.7%)	14	0.936 ± 0.026	0.132 ± 0.031	Successful runs (*n* = 13): 6 Unsuccessful runs (*n* = 2): 6	Successful runs (*n* = 13): 4 Unsuccessful runs (*n* = 2): 9	Two runs terminated after 3 retries due to noncompliance
Overall	30	28 (93.3%)	—	0.942 ± 0.025	0.125 ± 0.031	6	5	Each block had a minimum of 2 successful runs

Timeout, number of times that the model was not able to generate a schedule within 500 s; Jain Index, fairness index (0-1 higher = more equitable); Gini Index, inequity index (0-1, lower = more equitable); SD, standard deviation.

**Table 3.** Summary of Efficiency Metrics Across Successful Model Runs.

Category	Total Successful Runs	Wall-Clock Time (s) (Mean ± SD)	Attempt Time (s) (Mean ± SD)	Cost Per Run (United States Dollars) (Mean ± SD)
Nonbackup blocks	15	236.19 ± 147.69	202.85 ± 52.93	0.15 ± 0.03
Backup blocks	13	447.88 ± 400.06	255.57 ± 54.60	0.24 ± 0.08
Overall	28	334.47 ± 306.60	227.33 ± 59.16	0.19 ± 0.08

API, application programming interface; wall-clock time, elapsed duration from initiation to successful schedule; attempt time, duration of successful API call (resets after every 500 s timeout); Cost, OpenAI application programming interface token-based estimate for gpt-5.2.

### Backup Blocks

Among the 5 backup blocks, all 13 of 15 (86.7%) runs were successful in generating call schedules that adhered to all 14 rules, however each block (5 of 5; 100%) had at least 2 successful final schedules generated. For 4 of the successful runs, 1 retry was needed due to noncompliant schedule generation and for 1 run, 2 retries were needed. For 2 other successful runs, timeouts at the 500 s mark occurred twice requiring retries. For the 2 nonsuccessful attempts, 1 timed out 7 times and the script terminated due to 3 consecutive noncompliant schedules generated. Another run timed out twice and the script also terminated due to 3 consecutive noncompliant schedules generated. The median (range) Jain was 0.940 (0.080) with a mean (SD) of 0.936 (0.025). Furthermore, the median (range) Gini was 0.134 (0.098) with a mean of 0.132 (0.031).

The median (range) total wall-clock time for the 13 successful runs was 295.07 (1139.41) s while the mean (SD) was 447.88 (400.06) s. The median (range) time for individual successful attempts was 259.92 (161.04) s while the mean (SD) was 255.57 (54.7) s. The median (range) cost per run was $0.25 ($0.32) USD while the mean (SD) was $0.24 ($0.08) USD (Tables 2 and 3). All outputs can be found in the Online Supplementary Material.

## Discussion

The primary finding of this article was the GPT-5.2 combined with a deterministic Python rule-checker, can rapidly generate rule-compliant, equitable orthopedic resident call schedules with minimal retries and low costs per schedule. This study only accepted schedules if they satisfied 14 complex constraints (eg, adhering to vacation and “fly-in” dates, specific weekend definitions, eligibility for backup calls, and equity within-PGY cohorts and across multiple PGY cohorts), suggesting that LLMs in its current form have the potential to dramatically decrease the administrative burden with scheduling.

Historically, classic operations-research approaches (eg, mixed integer programming, constraint programming, and optimization-based algorithms) have been proposed for healthcare workforce scheduling tasks, such as scheduling residents. Mixed integer programming models real-world scheduling problems mathematically with decision variables, complex constraints, and objective functions and aims to optimize a schedule.^13^ For example, a previous study formulated an integer programming model to schedule 56 residents across 4 hospitals, 11 services, and 42 shifts under an 80-h work week.^3^ While the model produced a schedule that satisfied all constraints, the reported computer run-time was 25.9 h.^3^ Another study reported that even after adopting an automated scheduling system, the total run-time to schedule shifts within an emergency department for a month ranged between 4 and 6 h.^14^ Collectively, these studies suggest that optimization can reduce manual burden, but that classic optimization algorithms may still remain a multihour task. In contrast, this current study suggests that night-call scheduling can be completed within a time-span of minutes when using LLMs. Additionally, while integer programming can tolerate a high degree of constraints, practical deployment across a wide range of institutions requires translation of local policies into specific mathematical formulae. In contrast, an LLM-based approach can more rapidly adapt to local rules due to prompt-level changes in English text, with hard code only needed for rule validation.

The concept of using LLMs for scheduling within healthcare has largely been unexplored. One recent study utilized ChatGPT-4 to schedule calls for 5 ophthalmology residents.^15^ The rules that the model was required to follow included: only one resident can be assigned per day, the same resident works Friday and Sunday, the same resident never works 2 weekdays in a single week, residents cannot work 2 days consecutively, residents cannot work if they worked 2 days prior, residents cannot work 2 weekends in a row, and residents cannot work on unavailable dates.^15^ The current study builds on the results from this, using a stronger model (gpt-5.2) to schedule residents between 10 blocks as opposed to just 1. Furthermore, the current study forces the model to be compliant to double the amount of rules than the previous paper, and also includes a higher degree of complexity by including scenarios where juniors need to be backed up by select senior residents. Additionally, despite the natural hierarchy that exists within a residency program, where senior residents take less call than junior residents, the Jain scores ranged between 0.897 and 0.989 and the Gini ranged between 0.05 and 0.177, suggesting a high degree of “fairness.”

The rapid progression of LLM tools offer tremendous potential in the ability to reduce the amount of administrative burden faced by clinicians.^7,16-18^ Scheduling often is performed by a designated chief resident, one previous study noted that their chief resident may spend hours on schedule creation, and despite this, the final generated schedule may have a mixed effect on the overall satisfaction by resident bodies.^2^ A previous survey of chief residents in an emergency medicine department found that they spend up to 7.9 h a week on administrative tasks including scheduling.^19^ Automation with LLMs allow chief residents to have less burden and burnout, and allow for more time for alternative activities such as studying or patient care. Additionally, automation software allows chief residents to easily test if last minute vacation or away requests, or lengthier requests can be feasible from a coverage point of view. Studies demonstrating the validity of LLMs in performing various administrative tasks within healthcare are important in order to suggest viability in clinical use. For example, there are up to 90 platforms in existence to date for ambient scribes using AI, with several studies supporting the use of this technology for note-writing.^20-24^ Future studies are encouraged to continue to investigate the use of LLMs for automated scheduling, including scheduling of yearly rotations, daily assignments, and scheduling of calls with a different set of rules.

While this study represents one of the first implementations of a formal assessment of LLMs for fully automating complex, rule-compliant call schedules within surgery, there are a few limitations with this analysis. First, it was conducted within a single orthopedic surgery residency program, therefore the rule set utilized reflected local institutional policies and call structures. Vacation and away dates were selected to reflect those requested over previous blocks and an increased number of requested vacation days for future blocks can affect the model. The ability for GPT-5.2 to perform within the constraints of other programs, though likely, cannot be concluded based on these results. Second, the real-world applicability of this technology was not assessed as the study did not measure reductions in administrative workload for chief residents or user satisfaction with the generated call schedules. Third, as with all LLMs, there are limitations inherently related to server-based execution. In this study, there were occasional timeouts on certain runs occurring despite identical input files. For example, in 2 of the backup blocks, the model successfully generated 2 compliant schedules but timed out or produced repeated noncompliant outputs on the third attempt. The reason for this variability is unclear, but may relate to transient server latency, queue congestion, or the nondeterministic nature of using generative AI. Despite this, for all 10 blocks, at least 2 successful outputs were still generated. Finally, no formal sample size calculation was performed given the simulation-based methodological nature of the study. The number of simulated blocks was selected to reflect a range of realistic scheduling scenarios rather than to achieve statistical power.

## Conclusion

LLMs, specifically, GPT-5.2, can automate the generation of complex, rule-compliant and equitable call schedules for orthopedic surgery residents within minutes at a low computational cost of less than $1.00USD.

## Supplemental Material

sj-json-1-mde-10.1177_23821205261441470 - Supplemental material for Automating Rule-Compliant and Equitable Call Schedules for Orthopedic Surgery Residents With Artificial Intelligence and Large Language Models: A Simulation-Based Validation Study Supplemental material, sj-json-1-mde-10.1177_23821205261441470 for Automating Rule-Compliant and Equitable Call Schedules for Orthopedic Surgery Residents With Artificial Intelligence and Large Language Models: A Simulation-Based Validation Study by Prushoth Vivekanantha, Marc Daniel Bouchard, Jeffrey Kay, Darren de SA and Olufemi R. Ayeni in Journal of Medical Education and Curricular Development

sj-csv-2-mde-10.1177_23821205261441470 - Supplemental material for Automating Rule-Compliant and Equitable Call Schedules for Orthopedic Surgery Residents With Artificial Intelligence and Large Language Models: A Simulation-Based Validation Study Supplemental material, sj-csv-2-mde-10.1177_23821205261441470 for Automating Rule-Compliant and Equitable Call Schedules for Orthopedic Surgery Residents With Artificial Intelligence and Large Language Models: A Simulation-Based Validation Study by Prushoth Vivekanantha, Marc Daniel Bouchard, Jeffrey Kay, Darren de SA and Olufemi R. Ayeni in Journal of Medical Education and Curricular Development

sj-csv-3-mde-10.1177_23821205261441470 - Supplemental material for Automating Rule-Compliant and Equitable Call Schedules for Orthopedic Surgery Residents With Artificial Intelligence and Large Language Models: A Simulation-Based Validation Study Supplemental material, sj-csv-3-mde-10.1177_23821205261441470 for Automating Rule-Compliant and Equitable Call Schedules for Orthopedic Surgery Residents With Artificial Intelligence and Large Language Models: A Simulation-Based Validation Study by Prushoth Vivekanantha, Marc Daniel Bouchard, Jeffrey Kay, Darren de SA and Olufemi R. Ayeni in Journal of Medical Education and Curricular Development

sj-csv-4-mde-10.1177_23821205261441470 - Supplemental material for Automating Rule-Compliant and Equitable Call Schedules for Orthopedic Surgery Residents With Artificial Intelligence and Large Language Models: A Simulation-Based Validation Study Supplemental material, sj-csv-4-mde-10.1177_23821205261441470 for Automating Rule-Compliant and Equitable Call Schedules for Orthopedic Surgery Residents With Artificial Intelligence and Large Language Models: A Simulation-Based Validation Study by Prushoth Vivekanantha, Marc Daniel Bouchard, Jeffrey Kay, Darren de SA and Olufemi R. Ayeni in Journal of Medical Education and Curricular Development

sj-json-5-mde-10.1177_23821205261441470 - Supplemental material for Automating Rule-Compliant and Equitable Call Schedules for Orthopedic Surgery Residents With Artificial Intelligence and Large Language Models: A Simulation-Based Validation Study Supplemental material, sj-json-5-mde-10.1177_23821205261441470 for Automating Rule-Compliant and Equitable Call Schedules for Orthopedic Surgery Residents With Artificial Intelligence and Large Language Models: A Simulation-Based Validation Study by Prushoth Vivekanantha, Marc Daniel Bouchard, Jeffrey Kay, Darren de SA and Olufemi R. Ayeni in Journal of Medical Education and Curricular Development

sj-csv-6-mde-10.1177_23821205261441470 - Supplemental material for Automating Rule-Compliant and Equitable Call Schedules for Orthopedic Surgery Residents With Artificial Intelligence and Large Language Models: A Simulation-Based Validation Study Supplemental material, sj-csv-6-mde-10.1177_23821205261441470 for Automating Rule-Compliant and Equitable Call Schedules for Orthopedic Surgery Residents With Artificial Intelligence and Large Language Models: A Simulation-Based Validation Study by Prushoth Vivekanantha, Marc Daniel Bouchard, Jeffrey Kay, Darren de SA and Olufemi R. Ayeni in Journal of Medical Education and Curricular Development

sj-csv-7-mde-10.1177_23821205261441470 - Supplemental material for Automating Rule-Compliant and Equitable Call Schedules for Orthopedic Surgery Residents With Artificial Intelligence and Large Language Models: A Simulation-Based Validation Study Supplemental material, sj-csv-7-mde-10.1177_23821205261441470 for Automating Rule-Compliant and Equitable Call Schedules for Orthopedic Surgery Residents With Artificial Intelligence and Large Language Models: A Simulation-Based Validation Study by Prushoth Vivekanantha, Marc Daniel Bouchard, Jeffrey Kay, Darren de SA and Olufemi R. Ayeni in Journal of Medical Education and Curricular Development

sj-json-8-mde-10.1177_23821205261441470 - Supplemental material for Automating Rule-Compliant and Equitable Call Schedules for Orthopedic Surgery Residents With Artificial Intelligence and Large Language Models: A Simulation-Based Validation Study Supplemental material, sj-json-8-mde-10.1177_23821205261441470 for Automating Rule-Compliant and Equitable Call Schedules for Orthopedic Surgery Residents With Artificial Intelligence and Large Language Models: A Simulation-Based Validation Study by Prushoth Vivekanantha, Marc Daniel Bouchard, Jeffrey Kay, Darren de SA and Olufemi R. Ayeni in Journal of Medical Education and Curricular Development

sj-csv-9-mde-10.1177_23821205261441470 - Supplemental material for Automating Rule-Compliant and Equitable Call Schedules for Orthopedic Surgery Residents With Artificial Intelligence and Large Language Models: A Simulation-Based Validation Study Supplemental material, sj-csv-9-mde-10.1177_23821205261441470 for Automating Rule-Compliant and Equitable Call Schedules for Orthopedic Surgery Residents With Artificial Intelligence and Large Language Models: A Simulation-Based Validation Study by Prushoth Vivekanantha, Marc Daniel Bouchard, Jeffrey Kay, Darren de SA and Olufemi R. Ayeni in Journal of Medical Education and Curricular Development

sj-csv-10-mde-10.1177_23821205261441470 - Supplemental material for Automating Rule-Compliant and Equitable Call Schedules for Orthopedic Surgery Residents With Artificial Intelligence and Large Language Models: A Simulation-Based Validation Study Supplemental material, sj-csv-10-mde-10.1177_23821205261441470 for Automating Rule-Compliant and Equitable Call Schedules for Orthopedic Surgery Residents With Artificial Intelligence and Large Language Models: A Simulation-Based Validation Study by Prushoth Vivekanantha, Marc Daniel Bouchard, Jeffrey Kay, Darren de SA and Olufemi R. Ayeni in Journal of Medical Education and Curricular Development

sj-json-11-mde-10.1177_23821205261441470 - Supplemental material for Automating Rule-Compliant and Equitable Call Schedules for Orthopedic Surgery Residents With Artificial Intelligence and Large Language Models: A Simulation-Based Validation Study Supplemental material, sj-json-11-mde-10.1177_23821205261441470 for Automating Rule-Compliant and Equitable Call Schedules for Orthopedic Surgery Residents With Artificial Intelligence and Large Language Models: A Simulation-Based Validation Study by Prushoth Vivekanantha, Marc Daniel Bouchard, Jeffrey Kay, Darren de SA and Olufemi R. Ayeni in Journal of Medical Education and Curricular Development

sj-csv-12-mde-10.1177_23821205261441470 - Supplemental material for Automating Rule-Compliant and Equitable Call Schedules for Orthopedic Surgery Residents With Artificial Intelligence and Large Language Models: A Simulation-Based Validation Study Supplemental material, sj-csv-12-mde-10.1177_23821205261441470 for Automating Rule-Compliant and Equitable Call Schedules for Orthopedic Surgery Residents With Artificial Intelligence and Large Language Models: A Simulation-Based Validation Study by Prushoth Vivekanantha, Marc Daniel Bouchard, Jeffrey Kay, Darren de SA and Olufemi R. Ayeni in Journal of Medical Education and Curricular Development

sj-csv-13-mde-10.1177_23821205261441470 - Supplemental material for Automating Rule-Compliant and Equitable Call Schedules for Orthopedic Surgery Residents With Artificial Intelligence and Large Language Models: A Simulation-Based Validation Study Supplemental material, sj-csv-13-mde-10.1177_23821205261441470 for Automating Rule-Compliant and Equitable Call Schedules for Orthopedic Surgery Residents With Artificial Intelligence and Large Language Models: A Simulation-Based Validation Study by Prushoth Vivekanantha, Marc Daniel Bouchard, Jeffrey Kay, Darren de SA and Olufemi R. Ayeni in Journal of Medical Education and Curricular Development

sj-csv-14-mde-10.1177_23821205261441470 - Supplemental material for Automating Rule-Compliant and Equitable Call Schedules for Orthopedic Surgery Residents With Artificial Intelligence and Large Language Models: A Simulation-Based Validation Study Supplemental material, sj-csv-14-mde-10.1177_23821205261441470 for Automating Rule-Compliant and Equitable Call Schedules for Orthopedic Surgery Residents With Artificial Intelligence and Large Language Models: A Simulation-Based Validation Study by Prushoth Vivekanantha, Marc Daniel Bouchard, Jeffrey Kay, Darren de SA and Olufemi R. Ayeni in Journal of Medical Education and Curricular Development

sj-json-15-mde-10.1177_23821205261441470 - Supplemental material for Automating Rule-Compliant and Equitable Call Schedules for Orthopedic Surgery Residents With Artificial Intelligence and Large Language Models: A Simulation-Based Validation Study Supplemental material, sj-json-15-mde-10.1177_23821205261441470 for Automating Rule-Compliant and Equitable Call Schedules for Orthopedic Surgery Residents With Artificial Intelligence and Large Language Models: A Simulation-Based Validation Study by Prushoth Vivekanantha, Marc Daniel Bouchard, Jeffrey Kay, Darren de SA and Olufemi R. Ayeni in Journal of Medical Education and Curricular Development

sj-csv-16-mde-10.1177_23821205261441470 - Supplemental material for Automating Rule-Compliant and Equitable Call Schedules for Orthopedic Surgery Residents With Artificial Intelligence and Large Language Models: A Simulation-Based Validation Study Supplemental material, sj-csv-16-mde-10.1177_23821205261441470 for Automating Rule-Compliant and Equitable Call Schedules for Orthopedic Surgery Residents With Artificial Intelligence and Large Language Models: A Simulation-Based Validation Study by Prushoth Vivekanantha, Marc Daniel Bouchard, Jeffrey Kay, Darren de SA and Olufemi R. Ayeni in Journal of Medical Education and Curricular Development

sj-csv-17-mde-10.1177_23821205261441470 - Supplemental material for Automating Rule-Compliant and Equitable Call Schedules for Orthopedic Surgery Residents With Artificial Intelligence and Large Language Models: A Simulation-Based Validation Study Supplemental material, sj-csv-17-mde-10.1177_23821205261441470 for Automating Rule-Compliant and Equitable Call Schedules for Orthopedic Surgery Residents With Artificial Intelligence and Large Language Models: A Simulation-Based Validation Study by Prushoth Vivekanantha, Marc Daniel Bouchard, Jeffrey Kay, Darren de SA and Olufemi R. Ayeni in Journal of Medical Education and Curricular Development

sj-csv-18-mde-10.1177_23821205261441470 - Supplemental material for Automating Rule-Compliant and Equitable Call Schedules for Orthopedic Surgery Residents With Artificial Intelligence and Large Language Models: A Simulation-Based Validation Study Supplemental material, sj-csv-18-mde-10.1177_23821205261441470 for Automating Rule-Compliant and Equitable Call Schedules for Orthopedic Surgery Residents With Artificial Intelligence and Large Language Models: A Simulation-Based Validation Study by Prushoth Vivekanantha, Marc Daniel Bouchard, Jeffrey Kay, Darren de SA and Olufemi R. Ayeni in Journal of Medical Education and Curricular Development

sj-json-19-mde-10.1177_23821205261441470 - Supplemental material for Automating Rule-Compliant and Equitable Call Schedules for Orthopedic Surgery Residents With Artificial Intelligence and Large Language Models: A Simulation-Based Validation Study Supplemental material, sj-json-19-mde-10.1177_23821205261441470 for Automating Rule-Compliant and Equitable Call Schedules for Orthopedic Surgery Residents With Artificial Intelligence and Large Language Models: A Simulation-Based Validation Study by Prushoth Vivekanantha, Marc Daniel Bouchard, Jeffrey Kay, Darren de SA and Olufemi R. Ayeni in Journal of Medical Education and Curricular Development

sj-csv-20-mde-10.1177_23821205261441470 - Supplemental material for Automating Rule-Compliant and Equitable Call Schedules for Orthopedic Surgery Residents With Artificial Intelligence and Large Language Models: A Simulation-Based Validation Study Supplemental material, sj-csv-20-mde-10.1177_23821205261441470 for Automating Rule-Compliant and Equitable Call Schedules for Orthopedic Surgery Residents With Artificial Intelligence and Large Language Models: A Simulation-Based Validation Study by Prushoth Vivekanantha, Marc Daniel Bouchard, Jeffrey Kay, Darren de SA and Olufemi R. Ayeni in Journal of Medical Education and Curricular Development

sj-csv-21-mde-10.1177_23821205261441470 - Supplemental material for Automating Rule-Compliant and Equitable Call Schedules for Orthopedic Surgery Residents With Artificial Intelligence and Large Language Models: A Simulation-Based Validation Study Supplemental material, sj-csv-21-mde-10.1177_23821205261441470 for Automating Rule-Compliant and Equitable Call Schedules for Orthopedic Surgery Residents With Artificial Intelligence and Large Language Models: A Simulation-Based Validation Study by Prushoth Vivekanantha, Marc Daniel Bouchard, Jeffrey Kay, Darren de SA and Olufemi R. Ayeni in Journal of Medical Education and Curricular Development

sj-csv-22-mde-10.1177_23821205261441470 - Supplemental material for Automating Rule-Compliant and Equitable Call Schedules for Orthopedic Surgery Residents With Artificial Intelligence and Large Language Models: A Simulation-Based Validation Study Supplemental material, sj-csv-22-mde-10.1177_23821205261441470 for Automating Rule-Compliant and Equitable Call Schedules for Orthopedic Surgery Residents With Artificial Intelligence and Large Language Models: A Simulation-Based Validation Study by Prushoth Vivekanantha, Marc Daniel Bouchard, Jeffrey Kay, Darren de SA and Olufemi R. Ayeni in Journal of Medical Education and Curricular Development

sj-json-23-mde-10.1177_23821205261441470 - Supplemental material for Automating Rule-Compliant and Equitable Call Schedules for Orthopedic Surgery Residents With Artificial Intelligence and Large Language Models: A Simulation-Based Validation Study Supplemental material, sj-json-23-mde-10.1177_23821205261441470 for Automating Rule-Compliant and Equitable Call Schedules for Orthopedic Surgery Residents With Artificial Intelligence and Large Language Models: A Simulation-Based Validation Study by Prushoth Vivekanantha, Marc Daniel Bouchard, Jeffrey Kay, Darren de SA and Olufemi R. Ayeni in Journal of Medical Education and Curricular Development

sj-csv-24-mde-10.1177_23821205261441470 - Supplemental material for Automating Rule-Compliant and Equitable Call Schedules for Orthopedic Surgery Residents With Artificial Intelligence and Large Language Models: A Simulation-Based Validation Study Supplemental material, sj-csv-24-mde-10.1177_23821205261441470 for Automating Rule-Compliant and Equitable Call Schedules for Orthopedic Surgery Residents With Artificial Intelligence and Large Language Models: A Simulation-Based Validation Study by Prushoth Vivekanantha, Marc Daniel Bouchard, Jeffrey Kay, Darren de SA and Olufemi R. Ayeni in Journal of Medical Education and Curricular Development

sj-csv-25-mde-10.1177_23821205261441470 - Supplemental material for Automating Rule-Compliant and Equitable Call Schedules for Orthopedic Surgery Residents With Artificial Intelligence and Large Language Models: A Simulation-Based Validation Study Supplemental material, sj-csv-25-mde-10.1177_23821205261441470 for Automating Rule-Compliant and Equitable Call Schedules for Orthopedic Surgery Residents With Artificial Intelligence and Large Language Models: A Simulation-Based Validation Study by Prushoth Vivekanantha, Marc Daniel Bouchard, Jeffrey Kay, Darren de SA and Olufemi R. Ayeni in Journal of Medical Education and Curricular Development

sj-csv-26-mde-10.1177_23821205261441470 - Supplemental material for Automating Rule-Compliant and Equitable Call Schedules for Orthopedic Surgery Residents With Artificial Intelligence and Large Language Models: A Simulation-Based Validation Study Supplemental material, sj-csv-26-mde-10.1177_23821205261441470 for Automating Rule-Compliant and Equitable Call Schedules for Orthopedic Surgery Residents With Artificial Intelligence and Large Language Models: A Simulation-Based Validation Study by Prushoth Vivekanantha, Marc Daniel Bouchard, Jeffrey Kay, Darren de SA and Olufemi R. Ayeni in Journal of Medical Education and Curricular Development

sj-json-27-mde-10.1177_23821205261441470 - Supplemental material for Automating Rule-Compliant and Equitable Call Schedules for Orthopedic Surgery Residents With Artificial Intelligence and Large Language Models: A Simulation-Based Validation Study Supplemental material, sj-json-27-mde-10.1177_23821205261441470 for Automating Rule-Compliant and Equitable Call Schedules for Orthopedic Surgery Residents With Artificial Intelligence and Large Language Models: A Simulation-Based Validation Study by Prushoth Vivekanantha, Marc Daniel Bouchard, Jeffrey Kay, Darren de SA and Olufemi R. Ayeni in Journal of Medical Education and Curricular Development

sj-csv-28-mde-10.1177_23821205261441470 - Supplemental material for Automating Rule-Compliant and Equitable Call Schedules for Orthopedic Surgery Residents With Artificial Intelligence and Large Language Models: A Simulation-Based Validation Study Supplemental material, sj-csv-28-mde-10.1177_23821205261441470 for Automating Rule-Compliant and Equitable Call Schedules for Orthopedic Surgery Residents With Artificial Intelligence and Large Language Models: A Simulation-Based Validation Study by Prushoth Vivekanantha, Marc Daniel Bouchard, Jeffrey Kay, Darren de SA and Olufemi R. Ayeni in Journal of Medical Education and Curricular Development

sj-csv-29-mde-10.1177_23821205261441470 - Supplemental material for Automating Rule-Compliant and Equitable Call Schedules for Orthopedic Surgery Residents With Artificial Intelligence and Large Language Models: A Simulation-Based Validation Study Supplemental material, sj-csv-29-mde-10.1177_23821205261441470 for Automating Rule-Compliant and Equitable Call Schedules for Orthopedic Surgery Residents With Artificial Intelligence and Large Language Models: A Simulation-Based Validation Study by Prushoth Vivekanantha, Marc Daniel Bouchard, Jeffrey Kay, Darren de SA and Olufemi R. Ayeni in Journal of Medical Education and Curricular Development

sj-csv-30-mde-10.1177_23821205261441470 - Supplemental material for Automating Rule-Compliant and Equitable Call Schedules for Orthopedic Surgery Residents With Artificial Intelligence and Large Language Models: A Simulation-Based Validation Study Supplemental material, sj-csv-30-mde-10.1177_23821205261441470 for Automating Rule-Compliant and Equitable Call Schedules for Orthopedic Surgery Residents With Artificial Intelligence and Large Language Models: A Simulation-Based Validation Study by Prushoth Vivekanantha, Marc Daniel Bouchard, Jeffrey Kay, Darren de SA and Olufemi R. Ayeni in Journal of Medical Education and Curricular Development

sj-json-31-mde-10.1177_23821205261441470 - Supplemental material for Automating Rule-Compliant and Equitable Call Schedules for Orthopedic Surgery Residents With Artificial Intelligence and Large Language Models: A Simulation-Based Validation Study Supplemental material, sj-json-31-mde-10.1177_23821205261441470 for Automating Rule-Compliant and Equitable Call Schedules for Orthopedic Surgery Residents With Artificial Intelligence and Large Language Models: A Simulation-Based Validation Study by Prushoth Vivekanantha, Marc Daniel Bouchard, Jeffrey Kay, Darren de SA and Olufemi R. Ayeni in Journal of Medical Education and Curricular Development

sj-csv-32-mde-10.1177_23821205261441470 - Supplemental material for Automating Rule-Compliant and Equitable Call Schedules for Orthopedic Surgery Residents With Artificial Intelligence and Large Language Models: A Simulation-Based Validation Study Supplemental material, sj-csv-32-mde-10.1177_23821205261441470 for Automating Rule-Compliant and Equitable Call Schedules for Orthopedic Surgery Residents With Artificial Intelligence and Large Language Models: A Simulation-Based Validation Study by Prushoth Vivekanantha, Marc Daniel Bouchard, Jeffrey Kay, Darren de SA and Olufemi R. Ayeni in Journal of Medical Education and Curricular Development

sj-csv-33-mde-10.1177_23821205261441470 - Supplemental material for Automating Rule-Compliant and Equitable Call Schedules for Orthopedic Surgery Residents With Artificial Intelligence and Large Language Models: A Simulation-Based Validation Study Supplemental material, sj-csv-33-mde-10.1177_23821205261441470 for Automating Rule-Compliant and Equitable Call Schedules for Orthopedic Surgery Residents With Artificial Intelligence and Large Language Models: A Simulation-Based Validation Study by Prushoth Vivekanantha, Marc Daniel Bouchard, Jeffrey Kay, Darren de SA and Olufemi R. Ayeni in Journal of Medical Education and Curricular Development

sj-csv-34-mde-10.1177_23821205261441470 - Supplemental material for Automating Rule-Compliant and Equitable Call Schedules for Orthopedic Surgery Residents With Artificial Intelligence and Large Language Models: A Simulation-Based Validation Study Supplemental material, sj-csv-34-mde-10.1177_23821205261441470 for Automating Rule-Compliant and Equitable Call Schedules for Orthopedic Surgery Residents With Artificial Intelligence and Large Language Models: A Simulation-Based Validation Study by Prushoth Vivekanantha, Marc Daniel Bouchard, Jeffrey Kay, Darren de SA and Olufemi R. Ayeni in Journal of Medical Education and Curricular Development

sj-json-35-mde-10.1177_23821205261441470 - Supplemental material for Automating Rule-Compliant and Equitable Call Schedules for Orthopedic Surgery Residents With Artificial Intelligence and Large Language Models: A Simulation-Based Validation Study Supplemental material, sj-json-35-mde-10.1177_23821205261441470 for Automating Rule-Compliant and Equitable Call Schedules for Orthopedic Surgery Residents With Artificial Intelligence and Large Language Models: A Simulation-Based Validation Study by Prushoth Vivekanantha, Marc Daniel Bouchard, Jeffrey Kay, Darren de SA and Olufemi R. Ayeni in Journal of Medical Education and Curricular Development

sj-csv-36-mde-10.1177_23821205261441470 - Supplemental material for Automating Rule-Compliant and Equitable Call Schedules for Orthopedic Surgery Residents With Artificial Intelligence and Large Language Models: A Simulation-Based Validation Study Supplemental material, sj-csv-36-mde-10.1177_23821205261441470 for Automating Rule-Compliant and Equitable Call Schedules for Orthopedic Surgery Residents With Artificial Intelligence and Large Language Models: A Simulation-Based Validation Study by Prushoth Vivekanantha, Marc Daniel Bouchard, Jeffrey Kay, Darren de SA and Olufemi R. Ayeni in Journal of Medical Education and Curricular Development

sj-csv-37-mde-10.1177_23821205261441470 - Supplemental material for Automating Rule-Compliant and Equitable Call Schedules for Orthopedic Surgery Residents With Artificial Intelligence and Large Language Models: A Simulation-Based Validation Study Supplemental material, sj-csv-37-mde-10.1177_23821205261441470 for Automating Rule-Compliant and Equitable Call Schedules for Orthopedic Surgery Residents With Artificial Intelligence and Large Language Models: A Simulation-Based Validation Study by Prushoth Vivekanantha, Marc Daniel Bouchard, Jeffrey Kay, Darren de SA and Olufemi R. Ayeni in Journal of Medical Education and Curricular Development

sj-csv-38-mde-10.1177_23821205261441470 - Supplemental material for Automating Rule-Compliant and Equitable Call Schedules for Orthopedic Surgery Residents With Artificial Intelligence and Large Language Models: A Simulation-Based Validation Study Supplemental material, sj-csv-38-mde-10.1177_23821205261441470 for Automating Rule-Compliant and Equitable Call Schedules for Orthopedic Surgery Residents With Artificial Intelligence and Large Language Models: A Simulation-Based Validation Study by Prushoth Vivekanantha, Marc Daniel Bouchard, Jeffrey Kay, Darren de SA and Olufemi R. Ayeni in Journal of Medical Education and Curricular Development
